# Diterpenoid Lactones with Anti-Inflammatory Effects from the Aerial Parts of *Andrographis paniculata*

**DOI:** 10.3390/molecules24152726

**Published:** 2019-07-26

**Authors:** Lin Gan, Yuanru Zheng, Lijuan Deng, Pinghua Sun, Jiaxi Ye, Xiduan Wei, Feifei Liu, Linzhong Yu, Wencai Ye, Chunlin Fan, Junshan Liu, Wenqing Zhang

**Affiliations:** 1 Division of Cell, Developmental and Integrative Biology, School of Medicine, South China University of Technology, Guangzhou 510641, China; 2 School of Traditional Chinese Medicine, Southern Medical University, Guangzhou 510515, China; 3 Formula-pattern Research Center, School of Traditional Chinese Medicine, Jinan University, Guangzhou 510632, China; 4 Guangdong Province Key Laboratory of Pharmacodynamic Constituents of TCM and New Drugs Research, College of Pharmacy, Jinan University, Guangzhou 510632, China; 5 School of Traditional Chinese Pharmacy, China Pharmaceutical University, Nanjing 210009, China

**Keywords:** *Andrographis paniculate*, diterpenoid lactones, anti-inflammatory, 3D-QSAR

## Abstract

*Andrographis paniculata* (AP) has been widely used in China for centuries to treat various diseases, and especially to treat inflammation. Diterpenoid lactones are the main anti-inflammatory components of AP. However, systematic chemical composition and biological activities, as well as key pharmacophores, of these diterpenoid lactones from AP have not yet been clearly understood. In this study, 17 diterpenoid lactones, including 2 new compounds, were identified by spectroscopic methods, and most of them attenuated the generation of TNF-α and IL-6 in LPS-induced RAW 274.7 cells examined by ELISA. Pharmacophores of diterpenoid lactones responsible for the anti-inflammatory activities were revealed based on the quantitative structure-activity relationship (QSAR) models. Moreover, new compounds (**AP-1** and **AP-4**) exerted anti-inflammatory activity in LPS microinjection-induced zebrafish, which might be correlated with the inhibition of the translocation of NF-κB p65 from cytoplasm to nucleus. Our study provides guidelines for future structure modification and rational drug design of diterpenoid lactones with anti-inflammatory properties in medical chemistry.

## 1. Introduction

**Figure 1:**
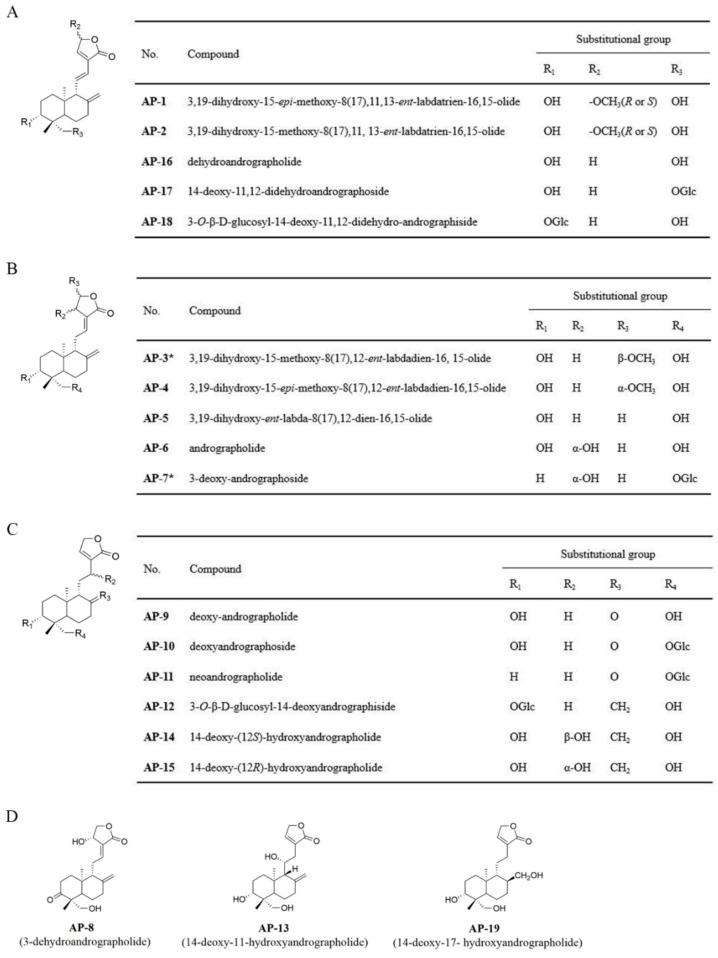
The chemical structures of 19 diterpenoid lactones (* compounds: published).

The aerial parts of *Andrographis paniculata* (Burm.f.) Nees (Acanthaceae) (AP) have been widely exploited in traditional medicine throughout China, India and other Southeast Asian countries [1,2]. Based on extensive research of the literature, modern pharmacological discoveries have greatly promoted the development of medical use of natural products [3,4]. In addition, it has demonstrated that AP contains various bioactive components, such as diterpene lactones, flavonoids and noriridoids [2,5,6] with immunestimulatory [7,8], anti-inflammatory [9,10], anticancer [11,12], antiangiogenic [13], antithrombotic [14], antidiabetic [15,16] and anti-oxidative effects [17]. Among them, diterpene lactones are considered the main biocomponents of AP, and they are extensively used to treat bronchitis, faucitis, pneumonia, amygdalitis such as Xiyanping injection and dripping pills of andrographolide [18,19,20]. Mechanism studies have demonstrated that andrographolide (Figure 1), the representative diterpene lactone of AP, shows significant anti-inflammatory properties by inhibiting NF-κB/MAPK activation [21,22] and the expression of Mac-1 [23]. However, current understanding of diterpenoid lactones from AP is still not enough, especially with regard to the systematic structure–activity relationship (SAR).

To enrich the diversity of natural diterpenoid lactones with better bioactivities, 17 diterpenoid lactones, including 2 new compounds, were isolated and identified from AP and the anti-inflammatory properties of these compounds were systemically evaluated in lipopolysaccharide (LPS)-stimulated RAW 264.7 macrophages. Thereby, we determined the key pharmacophores of diterpenoid lactones and developed a quantitative structure–activity relationship (QSAR) model for illustrating the relationship between the chemical structures and anti-inflammatory effects. Moreover, new compounds (**AP-1** and **AP-4**) inhibited the translocation of NF-κB p65 from cytoplasm to nucleus and attenuated inflammation in LPS-microinjection zebrafish. This research provides useful information for future structure modification and rational design of diterpenoid lactones as potent anti-inflammatory agents.

## 2. Results and Discussion

### 2.1. Identification of Isolated Diterpenoid Lactones

17 diterpenoid lactones were isolated and identified from the EtOAc fraction of AP, and the structures are exhibited in Figure 1, among which **AP-1** and **AP-4** are new compounds. The physicochemical properties and spectral data of **AP-1** and **AP-4** are depicted as follows, and the others were identified as 3,19-dihydroxy-15-methoxy-8(17),11, 13-*ent*-labdatrien-16,15-olide (**AP-2**) [24], 3,19-dihydroxy-*ent*-labda-8(17),12-dien-16,15-olide (**AP-5**) [24], andrographolide (**AP-6**) [25], 3-dehydroandrographolide (**AP-8**) [26], deoxy-andrographolide (**AP-9**) [25], deoxyandrographoside (**AP-10**) [25], neoandrographolide (**AP-11**) [25], 3-*O*-β-d-glucosyl-14-deoxyandrographiside (**AP-12**) [27], 14-deoxy-11-hydroxyandrographolide (**AP-13**) [25], 14-deoxy-(12*S*)-hydroxyandrographolide (**AP-14**) [28], 14-deoxy-(12*R*)-hydroxyandrographolide (**AP-15**) [28], dehydroandrographolide (**AP-16**) [25], 14-deoxy-11,12-didehydroandrographoside (**AP-17**) [25], 3-*O*-β-d-glucosyl-14-deoxy-11,12-didehydroandrographiside (**AP-18**) [27], and 14-deoxy-17-hydroxyandrographolide (**AP-19**) [29].

Compound **AP-1** was isolated as amorphous powder, ${\left[\alpha \right]}_{D}^{25}$ = −2.4 (*c* = 0.14, MeOH). The molecular formula was deduced to be C_21_H_30_O_5_ by its quasi-molecular ion at *m*/*z* 385.1988 [M + Na]^+^ (calcd. for C_21_H_30_O_5_Na 385.1985). The UV spectrum of **AP-1** displayed absorption maxima at 203 and 260 nm. The IR absorption showed the characteristics of hydroxyl (3407 cm^−1^), carbonyl (1767 cm^−1^) and double bond (1644 cm^−1^). In conjunction with the analysis of ^1^H and ^13^C NMR spectra, **AP-1** showed the characteristic signals of *ent*-labdane diterpene, including an exocyclic methylene [δ_H_ 4.87 (1H, d, *J* = 1.5 Hz) and 4.72 (1H, d, *J* = 1.5 Hz); δ_C_ 109.3], a oxymethylene [δ_H_ 4.49 (1H, d, *J* = 10.0 Hz) and 3.69 (1H, d, *J* = 10.0 Hz); δ_C_ 64.7], two tertiary methyls [δ_H_ 1.54 (3H, s); δ_C_ 24.2 and δ_H_ 0.90 (3H, s); δ_C_ 16.5]. The NMR spectra also showed signals due to an *α*, *β*-unsaturated *γ*-lactone moiety [δ_H_ 7.26 (1H, m); δ_C_ 142.1, 133.1] and a 1,2-disubstituted (*E*)-configured bond [*δ*_H_ 7.28 (1H, s); δ_C_ 139.1 and *δ*_H_ 6.30 (1H, d, *J* = 15.0 Hz); δ_C_ 121.9]. In addition, the signals belonging to an oxymethine [δ_H_ 6.01 (1H, d, *J* = 15.0 Hz); δ_C_ 103.3] and a methoxy group [δ_H_ 3.50 (3H, s); δ_C_ 57.1] were also observed. All the above spectral data indicated that **AP-1** was an andrographolide analogue [25]. ^1^H NMR, ^13^C NMR, ^1^H-^1^H correlation spectroscopy (COSY), heteronuclear single quantum coherence spectroscopy (HSQC) and heteronuclear multiple-bond correlation spectroscopy (HMBC) signals of **AP-1** were assigned as shown in Table 1. The ^1^H NMR and ^13^C NMR data of **AP-1** were similar to those of **AP-2** [24], which implied that both of them shared the same planar structure.

**Figure 2:**
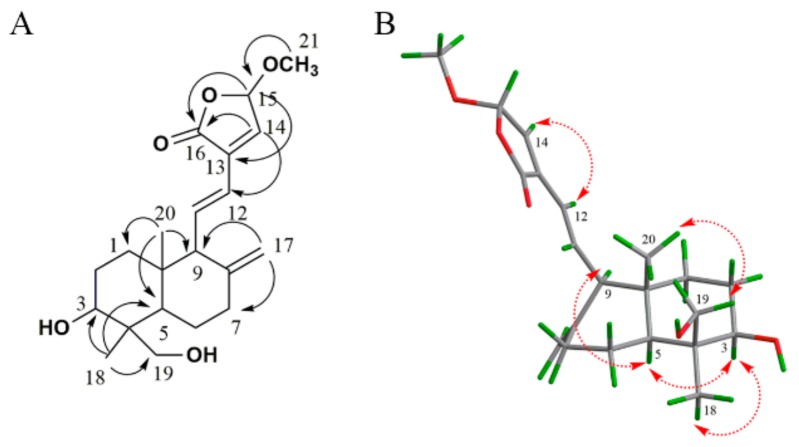
Key HMBC correlations (**A**) and key NOESY correlations (**B**) of **AP-1**.

Furthermore, HMBC correlations between H-14 (*δ*_H_ 7.26) and C-16 (*δ*_C_ 170.7), H-15 (*δ*_H_ 6.01) and C-13 (*δ*_C_ 133.1)/C-16 (*δ*_C_ 170.7), as well as H-14 (*δ*_H_ 7.26) and C-12 (*δ*_C_ 121.9) (Figure 2A), further confirmed that **AP-1** and **AP-2** shared the same planar structure. In the nuclear overhauser effect spectroscopy (NOESY) spectrum, s-transoid conformation of conjugated double bonds in **AP-1** was assigned according to the correlation of H-14 (*δ*_H_ 7.26) to H-12 (*δ*_H_ 6.30). Correlations between H-3 (*δ*_H_ 3.67) and H-5 (*δ*_H_ 1.23)/H_3_-18 (*δ*_H_ 1.54), H-19 (*δ*_H_ 4.49) and H_3_-20 (*δ*_H_ 0.90), and between H-5 (*δ*_H_ 1.23) and H-9 (*δ*_H_ 2.41) were also observed, indicating that H-3, H-5 and H-18 were on the same orientation, while H-11, H-19 and H-20 were displayed on the same orientation (Figure 2B, mixing time 300 ms). Taken together, compound **AP-1** was elucidated as 3,19-dihydroxy-15-*epi*-methoxy-8(17),11,13-*ent*-labdatrien-16,15-olide.

Compound **AP-4** was obtained as amorphous powder, ${\left[\alpha \right]}_{D}^{25}$ = −3.8 (*c* = 0.1, MeOH). The molecular formula of **AP-4** was determined as C_21_H_30_O_5_ from the molecular ion peak at *m*/*z* 387.2147 [M + Na]^+^ (calcd. for C_21_H_32_O_5_Na 387.2142). Similar to **AP-1**, compound **AP-4** displayed the characteristic UV (*λ*_max_ 203 and 225 nm) and IR (*v*_max_ 3395, 1757 and 1644 cm^−1^) absorptions for a diterpenoid lactone skeleton. The ^1^H and ^13^C NMR spectra of **AP-4** also revealed the presence of an *ent*-labdane diterpene with an *α*, *β*-unsaturated *γ*-lactone ring, including the signal due to a carbonyl group (*δ*_C_ 171.8), an exocyclic double band [*δ*_H_ 4.89 (1H, br s), 4.45 (1H, br s); *δ*_C_ 149.0, 108.5], an olefinic bond [*δ*_H_ 6.62 (1H, m); *δ*_C_ 143.6, 126.0], an oxygenated methylene [*δ*_H_ (4.11 (1H, d, *J* = 11.0 Hz), 3.36 (1H, d, *J* = 11.0 Hz); *δ*_C_ 65.0), and two tertiary methyls [*δ*_H_ 1.22 (3H, s); *δ*_C_ 23.4 and *δ*_H_ 0.73 (3H, s); *δ*_C_ 15.5]. Additionally, signals corresponding to an acetal group [*δ*_H_ 5.55 (1H, dd, *J* = 6.5, 2.0 Hz); *δ*_C_ 104.4], an oxymethine [*δ*_H_ 3.40 (1H, m); *δ*_C_ 80.9] and a methoxy group [*δ*_H_ 3.50 (3H, s); *δ*_C_ 57.2] were also observed. Compared with the spectroscopic data of **AP-3** [30], NMR data (Table 2) revealed they shared the same planar structure.

**Figure 3:**
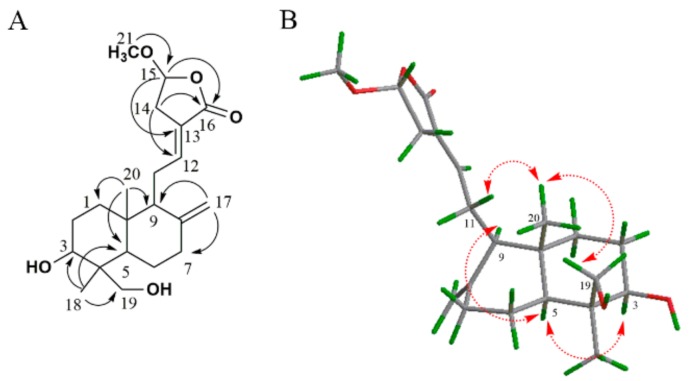
Key HMBC correlations (**A**) and key NOESY correlations (**B**) of **AP-4**.

Further comparison of HMBC correlations of H_3_-21 (*δ*_H_ 3.50) to C-15 (*δ*_C_ 104.4), H-15 (*δ*_H_ 5.55) to C-16 (*δ*_C_ 171.8) and C-13 (*δ*_C_ 126.0) (Figure 3A) clearly confirmed that **AP-3** and **AP-4** shared the same planar structure. In the NOESY spectrum, correlations between H-5 (*δ*_H_ 1.32) and H-3 (*δ*_H_ 3.40)/H-9 (*δ*_H_ 1.90), and between H-20 (*δ*_H_ 0.73) and H-19 (*δ*_H_ 4.11)/H-11 (*δ*_H_ 2.39) were observed, suggesting that H-3, H-5 and 18-CH_3_ were on the same orientation, while 19-CH_2_OH and 20-CH_3_ were displayed on the other orientation (Figure 3B). The opposite relative configuration between **AP-4** and **AP-3** indicated that H-15, H-3, H-5 and 18-CH_3_ were on the same orientation. Thus, the relative configuration of **AP-4** was further defined and named as 3,19-dihydroxy-15-*epi*-methoxy-8(17),12-*ent*-labdadien-16,15-olide.

### 2.2. Anti-Inflammatory Effects of 19 Diterpenoid Lactones in LPS-Induced RAW 264.7 Cells

We first investigated the cytotoxicities of 19 diterpenoid lactones in RAW 264.7 cells by 3-(4,5-dimethylthiazol-2-yl)-2,5-diphenyl-tetrazolium bromide (MTT) assay. Data demonstrated that these compounds (including 2 published compounds, **AP-3** and **AP-7**, [30]) at the concentration of 10 μM had no obvious cytotoxicity in RAW 264.7 cells (data not shown).

Pro-inflammatory cytokines interleukin-6 (IL-6) and tumor necrosis factor-α (TNF-α) play important roles in the inflammatory response [31,32]. LPS can stimulate the secretion of IL-6 and TNF-α in RAW 264.7 macrophage cells to induce inflammation [33]. To investigate the anti-inflammatory effects of isolated diterpenoid lactones, the levels of IL-6 and TNF-α in LPS-stimulated RAW 264.7 cells were determined. As shown in Table 3, most of compounds could significantly reduce the release of IL-6 and TNF-α. Moreover, **AP-6** and **AP-8** exerted the best activities, which were similar to that of dexamethasone (Dex, the positive control), indicating their potential as anti-inflammatory agents.

### 2.3. D-QSAR Analysis

**Figure 4:**
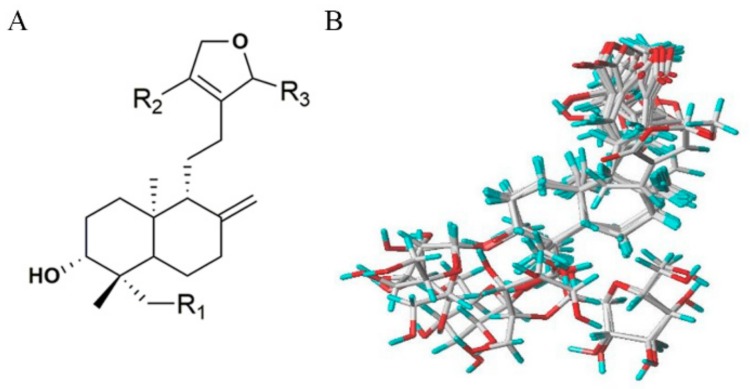
(**A**) The common skeleton for the alignment. (**B**) The alignment of all the compounds used in the data set.

Based on the anti-inflammatory effects and the chemical structures of 19 diterpenoid lactones, there is the possibility of developing an alignment rule for the superposition of the diverse derivatives to conduct the 3D-QSAR study by comparative molecular field analysis (CoMFA) and comparative molecular similarity indices analysis (CoMSIA) methods. Compound **AP-6**, with the greatest potential anti-inflammatory effect, was selected as the template for the alignment of all compounds. Figure 4A shows the common skeleton of 19 compounds, and the aligned molecules are shown in Figure 4B. The actual and predicted pI values of the training set and test set are listed in Table 4. The CoMFA and CoMSIA models gave good predictive correlation coefficients (*r*^2^_pred_) of 0.689 and 0.707, respectively, clearly suggesting that the models were reliable and capable of predicting the activity of diterpenoid lactones.

**Figure 5:**
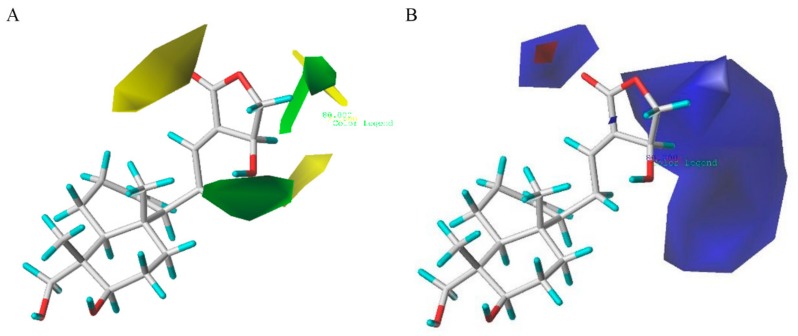
CoMFA Std*Coeff contour maps in combination with compound **AP-6** in group 1. (**A**) Steric field: green and yellow contours indicate regions where bulky groups enhance or reduce the activity, respectively. (**B**) Electrostatic field: blue and red contours indicate regions where electron-donating and electron-withdrawing groups enhance the potency, respectively.

**Figure 6:**
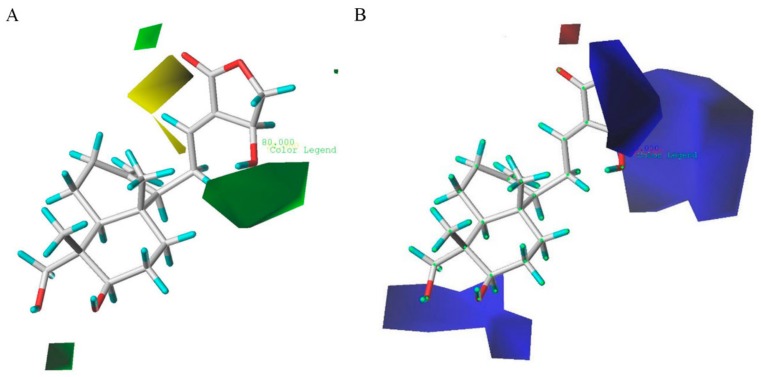
CoMFA Std*Coeff contour maps in combination with compound **AP-6** in group 2. (**A**) Steric field: green and yellow contours indicate regions where bulky groups enhance or reduce the activity, respectively. (**B**) Electrostatic field: blue and red contours indicate regions where electron-donating and electron-withdrawing groups enhance the potency, respectively.

The contour maps of CoMFA steric and electrostatic fields are depicted in Figure 5 (group 1: IL-6 group) and Figure 6 (group 2: TNF-α group), respectively. As shown in Figure 5A, the huge green contour near the R_2_ position suggested that a bulky group at this site would be beneficial for the activities. A small yellow contour near the R_3_ position indicated that a minor substituent at this site would be favorable. In Figure 5B, the huge blue contour above the R_2_ position indicated that an electron-donating group at this site would be favored. One blue contour close to the point of the R_3_ site also suggested that an electron-donating group at this position would be helpful for the activity. Figure 6A shows the contour maps of sterically favored (green) and disfavored (yellow) regions, which are similar to those in Figure 5A. In addition, the contour map in Figure 6B is also similar to that in Figure 5B. Additionally, a blue contour around the R_1_ position revealed that an electron-donating group at the distal part of R_1_ position was favorable. In addition, a small red contour can be observed around the R_3_ site, showing the substituent with electron-withdrawing was favored at this point.

**Figure 7:**
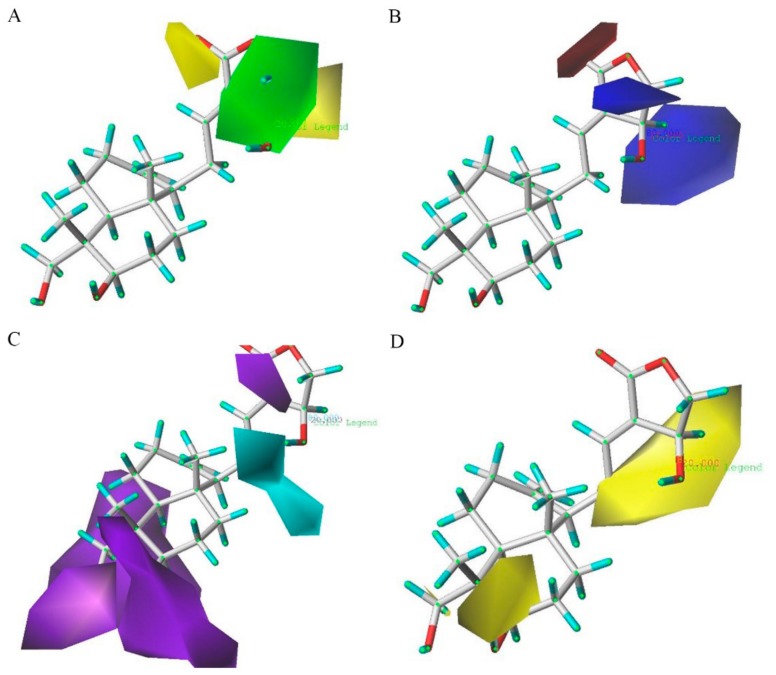
CoMSIA StDev*Coeff contour plots with the combination of compound **AP-6** in group 1. (**A**) Steric field: green and yellow contours refer to sterically favored and unfavored regions. (**B**) Electrostatic field: blue and red contours refer to regions where electron-donating and electron-withdrawing groups are favored, respectively. (**C**) Hydrogen bond donor field: the cyan and purple contours indicate favorable and unfavorable hydrogen bond donor groups. The contour plots of the hydrophobic field are depicted in figure (**D**); Hydrophobic field: yellow represents regions where hydrophobic groups increase activity; red represents regions where hydrophobic groups decrease activity.

**Figure 8:**
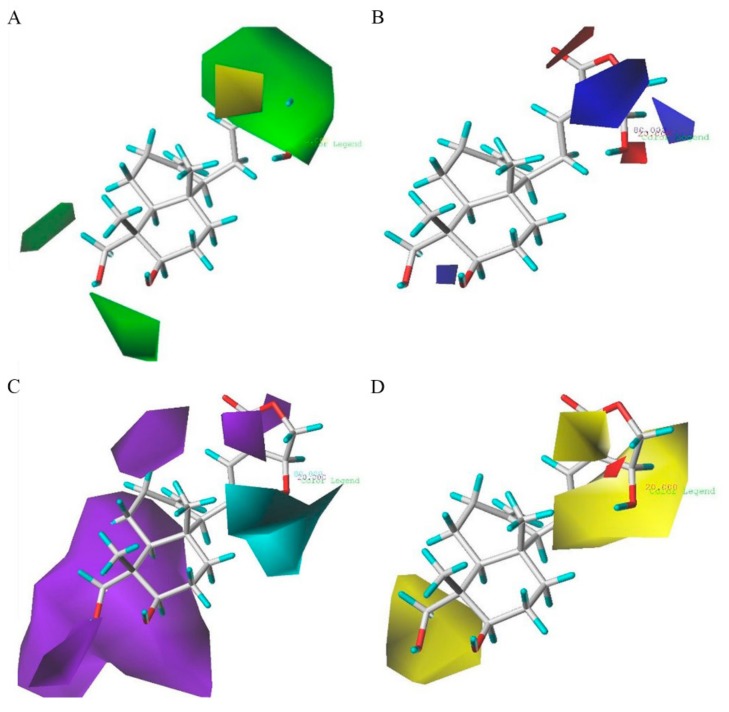
CoMSIA StDev*Coeff contour plots with the combination of compound **AP-6** in group 2. (**A**) Steric field: Green and yellow contours refer to sterically favored and unfavored regions. (**B**) Electrostatic field: Blue and red contours refer to regions where electron-donating and electron-withdrawing groups are favored, respectively. (**C**) Hydrogen bond donor field: The cyan and purple contours indicate favorable and unfavorable hydrogen bond donor groups. The contour plots of the hydrophobic field are depicted in figure (**D**); Hydrophobic field: Yellow represents regions where hydrophobic groups increase activity; red represents regions where hydrophobic groups decrease activity.

The CoMSIA steric, electrostatic, hydrogen bond donor and hydrophobic contours maps are demonstrated in Figure 7 (group 1) and Figure 8 (group 2). The CoMSIA steric and electrostatic contour maps in Figure 7A,B and Figure 8A,B are similar to the CoMFA contour maps shown in Figure 5 and Figure 6, respectively.

For the CoMSIA hydrogen bond donor field contour maps shown in Figure 7C, the cyan contours represent regions where hydrogen bond donor substituents were favorable for anti-inflammatory activity, whereas the purple contours were unfavorable. A huge purple contour along the R_1_ site indicates that the hydrogen bond acceptor field at this position was important for the activities. A small purple contour beside the R_3_ side chain shows that a hydrogen bond acceptor at this site would be favored. A cyan contour near the R_2_ position reveals that a hydrogen bond donor substituent at this position would increase the activity. The hydrogen bond donor fields of group 2 (Figure 8C) were basically similar to those of group 1 (Figure 7C).

The contour plots of the hydrophobic field are shown in Figure 7D and Figure 8D. The contour plots of the hydrophobic fields in group 1 are shown in Figure 7D, and these were in accordance with the results of group 2 (Figure 8D). In Figure 8D, there were large yellow regions near the substituents R_1_ and R_3_. This data indicates that the increased substituents in the regions would be of benefit to the biological activity. There is a huge yellow contour around the R_2_ position, demonstrating that hydrophobic groups play a critical role in this region.

**Figure 9:**
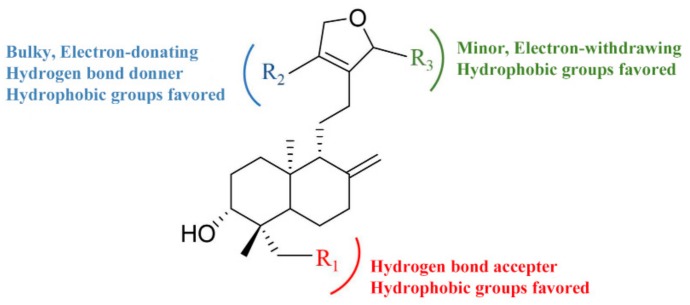
The structure-activity relationship of diterpenoid lactones revealed by 3D-QSAR study.

To sum up, the structure-activity relationship revealed by 3D-QSAR is illustrated in Figure 9. In detail, the hydrophobic and hydrogen bond acceptor groups at the R_1_ position are favorable; the bulky, electron-donating, hydrogen bond donner and hydrophobic groups at the R_2_ position, and the minor, electron-withdrawing hydrophobic groups at the R_3_ position, would increase the activity.

### 2.4. New Diterpenoid Lactones Inhibit the Activation of NF-Κb in LPS-Induced RAW 264.7 Cells

**Figure 10:**
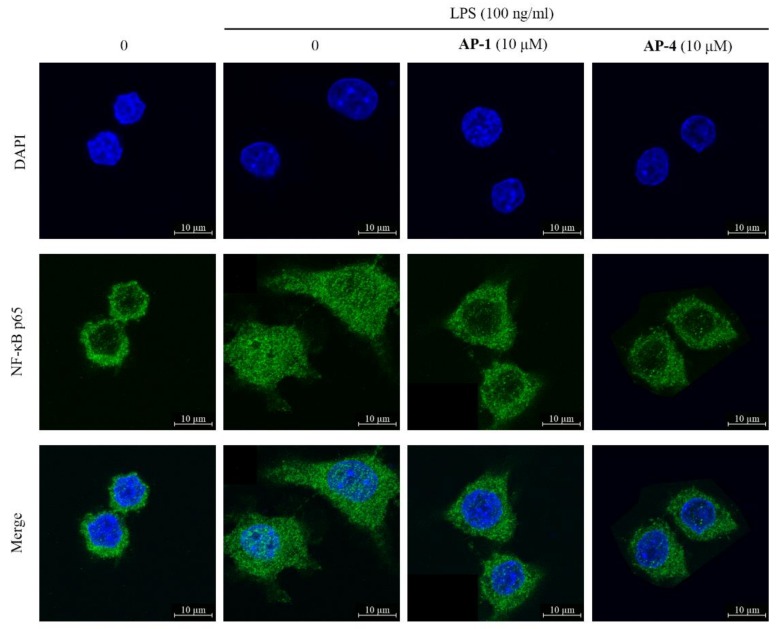
**AP-1** and **AP-4** prevented LPS-induced nuclear translocation of NF-κB p65 in RAW 264.7 macrophages. RAW 264.7 macrophage cells were pretreated with **AP-1** or **AP-4** (10 μM) for 2 h and incubated with or without LPS (100 ng/mL) for another 16 h. Then, cells were observed using a confocal microscope (400×, LSM880, Carl Zeiss, Germany).

The NF-κB signaling pathway is mainly responsible for the process of inflammatory and other diseases [34,35]. NF-κB p65, a critical regulatory transcription factor, separates from the inhibitory subunit when stimulation occurs and translocates into the nucleus, triggering the transcription of multiple inflammatory genes [36]. To research whether NF-κB participates in the anti-inflammatory process of new diterpenoid lactones (**AP-1** and **AP-4**), we tested the nuclear translocation of NF-κB p65. The results showed that **AP-1** and **AP-4** prevented the translocation of NF-κB p65 from cytoplasm to nucleus in LPS-stimulated RAW 264.7 macrophages (Figure 10), which indicated that **AP-1** and **AP-4** exert potent anti-inflammatory effects through the inhibition of NF-κB.

### 2.5. Anti-Inflammatory Effects of New Diterpenoid Lactones in Zebrafish

Zebrafish (*Danio rerio*) has gained tremendous attention as an animal model for assessing the anti-inflammatory effect due to their physiological similarity to mammals, small size, availability in large quantities, transparent body, and relatively low-cost maintenance [37,38]. LPS can induce systemic inflammation in zebrafish [39]. Considering that new diterpenoid lactones, **AP-1** and **AP-4** exhibited in vitro anti-inflammatory effects, we further evaluated in vivo anti-inflammatory activities by using an LPS-induced zebrafish model.

**Figure 11:**
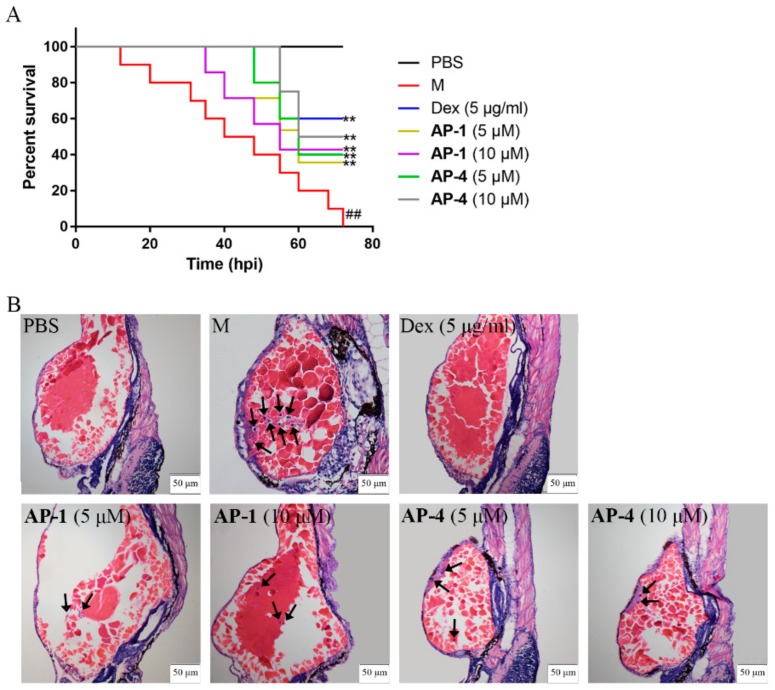
Protective effect of **AP-1** and **AP-4** in LPS-induced zebrafish. (**A**) **AP-1** and **AP-4** reduced the mortality of zebrafish after LPS injection. The yolks of larvae were microinjected with 2 mg/mL LPS at 3 days post fertilization (dpf), and mortality was monitored for 72 h. Negative controls were microinjected with phosphate-buffered saline (PBS). Data are represented as the mean of three independent experiments. ^##^ *p* < 0.01 versus control group, ** *p* < 0.01 versus model group by one-way ANOVA with Tukey’s test. (**B**) **AP-1** and **AP-4** attenuated the histopathological changes in LPS-stimulated zebrafish. The zebrafish were dehydrated and embedded in paraffin. Then, paraffin sections (4 μm) were stained by H&E. The black arrows represent inflammatory cells.

**Figure 12:**
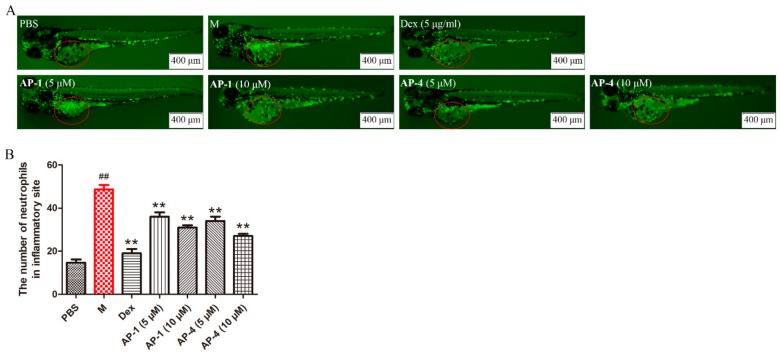
**AP-1** and **AP-4** alleviated the inflammatory response in zebrafish subjected to LPS microinjection. (**A**) Representative images of zebrafish in LPS-induced inflammation model. (**B**) Neutrophils in the region of interest (red circles) were quantitatively analyzed. Data are represented as the mean ± SE of three independent experiments. ^##^ *p* < 0.01 versus control group, ** *p* < 0.01 and * *p* < 0.05 versus model group by one-way ANOVA with Tukey’s test.

The survival rates showed that microinjection of LPS caused 100% mortality within 72 h, whereas **AP-1** and **AP-4** attenuated the mortality of zebrafish (Figure 11A). Infiltration of inflammatory cells is pivotal in the progression of inflammation [40]. Hematoxylin and eosin (H&E) staining results showed that inflammatory cell infiltration was clearly observed in the model group. However, the histopathological changes were ameliorated after **AP-1** and **AP-4** treatment (Figure 11B). Moreover, neutrophils significantly migrated and were recruited to the inflammatory site in the model group, while **AP-1** and **AP-4** reduced the number of neutrophils around the yolks, which is similar to Dex, as shown in Figure 12.

## 3. Materials and Methods

### 3.1. General Experimental Procedures

The experimental procedures were conducted as described previously [30].

### 3.2. Plant Material and Reagents

The dried ethanol extract of AP was provided by Sinopharm Group Dezhong (Foshan, Guangdong, China) Pharmaceutical Co., Ltd. (batch number: DZ130605). IL-6 and TNF-α ELISA kits were purchased from Dakewei (Beijing, China). MTT, dimethyl sulphoxide (DMSO) and LPS (*E. coli*, O55: B5, L2880, total impurities < 3% protein) were purchased from Sigma-Aldrich (St Louis, MO, USA).

### 3.3. Extraction and Isolation

The extraction and isolation procedures were performed as previously described [30]. The dried extract of AP (840 g) was suspended in H_2_O and successively partitioned with petroleum ether (PE), ethyl acetate (EtOAc) and *n*-butyl alcohol (*n*-BuOH). The extract was concentrated under vacuum to obtain PE fraction (30 g), EtOAc fraction (400 g), *n*-BuOH fraction (140 g) and water fraction, respectively. The EtOAc fraction (400 g) was subjected to silica gel column chromatography (CHCl_3_-MeOH, 100:0 to 0:100, *v*/*v*). Based on TLC profiles, similar fractions were combined to give eight fractions (Fr. 1 to Fr. 8). Fr. 2 was separated on a silica gel column, ODS column and preparative HPLC to yield **AP-5** (8 mg), **AP-9** (300 mg) and **AP-16** (300 mg). Fr. 3 (50 g) was subjected to a silica gel column with gradient conditions (CHCl_3_-MeOH, 100:0 to 0:100, *v*/*v*) to produce seven subfractions, which were further purified on a silica gel column, ODS column, preparative HPLC and recrystallized to give **AP-1** (15 mg), **AP-2** (15 mg), **AP-4** (15 mg), **AP-6** (5 g), **AP-8** (26 mg), **AP-13** (45 mg), **AP-14** (13 mg), **AP-15** (28 mg) and **AP-19** (15 mg). Similarly, Fr. 4 was separated on a silica gel column and preparative HPLC to collect **AP-11** (3 g). In addition, Fr. 5 was purified on a sephadex LH-20 column and preparative HPLC to afford **AP-10** (200 mg), **AP-12** (8 mg), **AP-17** (200 mg) and **AP-18** (20 mg).

### 3.4. Characterization of AP-1 and AP-4

**AP-1**: amorphous powder. ${\left[\alpha \right]}_{D}^{25}$ = −2.4 (*c* = 0.14, MeOH). UV (MeOH) *λ*_max_ 260, 203 nm. IR (KBr) 3407, 1767, 1644 cm^−1^. ^1^H NMR and ^13^C NMR data (Table 1). HRESIMS *m*/*z* 385.1988 [M + Na]^+^ (calcd. for C_21_H_30_O_5_Na 385.1985).

**AP-4**: amorphous powder. ${\left[\alpha \right]}_{D}^{25}$ = −3.8 (*c* = 0.1, MeOH). UV (MeOH) *λ*_max_ 225, 203 nm. IR (KBr) 3395, 1757, 1644 cm^−1^. ^1^H NMR and ^13^C NMR data (Table 2). HRESIMS *m*/*z* 387.2147 [M + Na]^+^ (calcd. for C_21_H_32_O_5_Na 387.2142).

### 3.5. Cell Culture

The murine macrophage cell line RAW 264.7 was purchased from the Cell Bank of the Chinese Academy of Sciences (Shanghai, China). Cells were cultured in DMEM supplemented with 10% (*v*/*v*) fetal bovine serum (Invitrogen) and 1% (*v*/*v*) penicillin/streptomycin (Gibco) at 37 °C in a humidified atmosphere with 5% CO_2_.

### 3.6. Cell Viability Assay

The cell viability was examined by MTT assay. RAW 264.7 cells were seeded in a 96-well plate at a density of 5 × 10^3^/well. Then, cells were treated with various concentrations of diterpenoid lactones for 24 h. Subsequently, 30 μL of MTT (5 mg/mL) was added into each well and incubated for another 4 h at 37 °C. The purple formazan crystals in each well were dissolved in 100 μL of DMSO and the absorbance was recorded at 570 nm by a microplate reader (Thermo Fisher Scientific, Waltham, MA, USA).

### 3.7. Enzyme-Linked Immunosorbent Assay (ELISA)

The levels of IL-6 and TNF-α in cell supernatants were quantified with ELISA kits. Firstly, the samples and Cytokine standard were diluted in a range of concentrations, and 100 μL/well was placed to the plate. After ninety-minute incubation at 37 °C, plates were washed with four times washing buffer, and Streptavidin-HRP was added to the wells at 100× dilution and incubated for another 30 min, and then washed again. Finally, TMB and Stop solution were added to measure the absorbance at 450 nm.

### 3.8. Confocal Microscopy

RAW 264.7 cells were cultured on a glass dish at a density of 1 × 10^5^ for 24 h. After being preincubated with **AP-1** (10 μM) or **AP-4** (10 μM) for 2 h, the following steps were carried out as previously described [41].

### 3.9. D-QSAR Modeling

To predict the potential anti-inflammatory effects of diterpenoid lactones, CoMFA and CoMSIA were performed. In group 1 (IL-6 group), 19 compounds were split randomly into training (15 compounds) and test sets (4 compounds), respectively. In addition, in group 2 (TNF-α group), 13 molecules were taken for the construction of training set, and the remaining 6 molecules were used to test the proposed CoMFA and CoMSIA models. The anti-inflammatory constants were expressed in pI values (pI = −log[the generation of pro-inflammatory cytokines after chemical treatment at the concentration of 10 μM]), which were applied as the dependent variables in CoMFA and CoMSIA models. In addition, the following experiments were performed as previously described [42].

### 3.10. Zebrafish Husbandry and Embryo Collection

The maintenance of adult transgenic zebrafish line Tg (MPO: GFP) was under standard conditions (Westerfield, 2000). Zebrafish were maintained under a 14-h light/10-h dark cycle at constant temperature (28 ± 0.5 °C) in an independent flow-through system (pH 7.2–7.6, salinity 0.03–0.04%). In addition, the zebrafish were fed brine shrimps three times a day.

Adult zebrafish were moved into a breeding tank until sufficient numbers of embryos were laid at the bottom of the tank. The embryos were washed and moved to clean Petri dishes filled with egg water containing 0.002% methylene blue (Sigma-Aldrich, St Louis, MO, USA) as a fungicide. Finally, these embryos were cultured in a warm oven (28.5 °C) for subsequent experiments.

### 3.11. LPS-Induced Inflammation Model and Chemical Treatments

Larvae were anesthetized at dpf in egg water containing 0.02% Tricaine (Sigma-Aldrich, St. Louis, MO, USA) and aligned in clear petri dishes coated with 1.5% agarose. Subsequently, the yolks were injected with 2 nL of LPS (2 mg/mL) to construct the lethal endotoxin-infected model [39] by using Cell Microinjector (PM1000, MicroData Instrument, Inc. S. Plainfield, NJ, USA). PBS served as the negative control. Then these larvae were washed with fresh egg water and were divided randomly into a 24-well plate (n = 20/well). The control group and the model group were treated with 2 mL of fresh egg water, and five drug groups were treated with Dex (5 μg/mL, Tianxin, Guangzhou, positive control), **AP-1** (5 μM), **AP-1** (10 μM), **AP-4** (5 μM) and **AP-4** (10 μM), respectively. For the survival experiment, egg water was changed and mortality (defined as lack of a discernible heart beat) was monitored daily. For the observation of neutrophils, the larvae were imaged at 12 h post LPS-microinjection using a fluorescence microscope (Olympus, MVX10, Tokyo, Japan).

### 3.12. Histopathological Examination of Zebrafish Larvae

Twelve hours after LPS-microinjection, the zebrafish were fixed immediately with 4% (*w*/*v*) paraformaldehyde overnight at room temperature, dehydrated in graded ethanol, embedded with paraffin (Leica, Wetzlar, Germany), and 4-µm-thick sections were cut. After being deparaffinized and stained with H&E (Yuanye Biotech, Shanghai, China), each section was observed under an IX 53 light microscope (Olympus, Tokyo, Japan).

### 3.13. Statistical Analysis

All the data are expressed as mean ± standard error (SE), and statistical analyses were performed using the software GraphPad Prism version 5.0 (GraphPad Prism Software, San Diego, CA, USA). Differences were statistically analyzed by Student’s *t*-test or ANOVA with Tukey’s test. *p* < 0.05 were considered statistically significant.

## 4. Conclusions

In conclusion, we isolated and identified 17 diterpenoid lactones from AP including 2 new compounds, most of the 19 diterpenoid lactones (including 2 published diterpenoid lactones) showed potent anti-inflammatory activities by reducing the secretion of IL-6 and TNF-α in LPS-stimulated RAW 264.7 macrophages. Furthermore, we discovered the key pharmacophores of diterpenoid lactones and developed a QSAR model to illustrate the significant relationship between the chemical structures and anti-inflammatory effects. Critically, the 2 new compounds exhibited significant anti-inflammatory activities in vitro and in vivo which might be correlated with the inhibition of the translocation of NF-κB p65 from cytoplasm to nucleus. Our study not only enriches the diversity of diterpenoid lactones in AP, but also provides guides for the further structural modification and rational drug design of diterpenoid lactones with anti-inflammatory activities in medical chemistry.

## Figures and Tables

**Table 1:** NMR data of **AP-1** (C_5_D_5_N, *δ*, *J* in Hz) ^1^.

No.	^1^H	^13^C	^1^H-^1^H COSY	HMBC	NOESY
1	1.45, 1.13 (m)	39.2	H-2	C-10, 20	−
2	1.95	29.4	H-1, 3	C-1, 3	−
3	3.67	80.6	H-2	C-19	H-1, 5, 18
4	−	43.9	−	−	−
5	1.23 (m)	55.2	H-6	C-6, 7, 20	H-3, 9
6	1.80 (m), 1.45	24.1	H-5, 7	C-7	−
7	2.41, 2.06	37.4	H-6	C-5, 9, 17	−
8	−	149.5	−	−	−
9	2.41	62.3	H-11	C-12, 17, 20	H-5, 7
10	−	39.6	−	−	−
11	7.28	139.1	H-9, 12	C-8, 13	−
12	6.30 (d, 15.0)	121.9	H-11	C-9, 14, 16	H-9, 14
13	−	133.1	−	−	−
14	7.26	142.1	H-15	C-12, 16	H-12, 17
15	6.01 (d, 15.0)	103.3	H-14	C-13, 16, 21	H-21
16	−	170.7	−	−	−
17	4.87 (d, 1.5), 4.72 (d, 1.5)	109.3	−	C-7, 9	H-7
18	1.54 (s)	24.2	−	C-3, 5, 19	H-3
19	4.49 (d, 10.0), 3.69 (d, 10.0)	64.7	−	C-3, 4, 5	H-2, 20
20	0.90 (s)	16.5	−	C-1, 5, 9	H-11, 19
21	3.50 (s)	57.1	−	C-15	−

^1^ Overlapped signals are reported without designating multiplicity.

**Table 2:** NMR data of **AP-4** (CD_3_OD, *δ*, *J* in Hz) ^1^.

No.	^1^H	^13^C	^1^H-^1^H COSY	HMBC	NOESY
1	1.79, 1.28	38.1	H-2	C-3, 5, 9, 20	−
2	1.79	29.0	H-1, 3	C-4,10	−
3	3.40	80.9	H-2	C-5, 18, 19	H-1, 5
4	−	43.7	−	−	−
5	1.32	56.3	H-6	C-18, 19, 20	H-3, 9
6	1.82, 1.37	25.2	H-5, 7	C-5, 7	−
7	2.43, 2.03 (m)	39.0	H-6	C-5, 6, 8, 17	−
8	−	149.0	−	−	−
9	1.90 (m)	57.0	H-11	C-12, 17, 20	H-1, 5
10	−	40.0	−	−	−
11	2.39, 2.27 (m)	26.6	H-9, 12	C-8, 12, 13	H-1, 7, 20
12	6.62 (m)	143.6	H-11	C-14, 16	H-9, 11
13	−	126.0	−	−	−
14	3.08 (m), 2.69 (m)	33.6	H-15	C-12, 16	H-11, 15
15	5.55 (dd, 6.5, 2.0)	104.4	H-14	C-14, 16, 21	H-14
16	−	171.8	−	−	−
17	4.89 (br s), 4.45 (br s)	108.5	−	C-7, 9	H-7
18	1.22 (s)	23.4	−	C-3, 5, 19	H-3
19	4.11 (d, 11.0), 3.36 (d, 11.0)	65.0	−	C-3, 5, 18	H-2, 20
20	0.73 (s)	15.5	−	C-1, 5, 9	H-11, 19
21	3.50 (s)	57.2	−	C-15	−

^1^ Overlapped signals are reported without designating multiplicity.

**Table 3:** Anti-inflammatory effects of 19 diterpenoid lactones on LPS-stimulated RAW 264.7 cells.

Compound	IL-6 (×10^−3^ mg)	TNF-α (×10^−3^ mg)	Compound	IL-6 (×10^−3^ mg)	TNF-α (×10^−3^ mg)
LPS (100 ng/mL)	1.36 ± 0.01	72.13 ± 2.89	**AP-10** (5 μM)	0.94 ± 0.04 **	66.64 ± 0.51 *
Dex (5 μM)	0.31 ± 0.02 **	32.21 ± 2.02 **	**AP-10** (10 μM)	0.94 ± 0.03 **	60.34 ± 2.75 **
**AP-1** (5 μM)	0.89 ± 0.02 **	63.77 ± 2.11 **	**AP-11** (5 μM)	1.13 ± 0.01 **	81.77 ± 5.00
**AP-1** (10 μM)	0.83 ± 0.03 **	53.00 ± 0.59 **	**AP-11** (10 μM)	1.11 ± 0.01 **	66.89 ± 3.23 *
**AP-2** (5 μM)	1.11 ± 0.03 **	62.72 ± 1.15 **	**AP-12** (5 μM)	0.93 ± 0.01 **	71.07 ± 3.03
**AP-2** (10 μM)	0.69 ± 0.01 **	57.05 ± 0.45 **	**AP-12** (10 μM)	0.81 ± 0.01 **	62.33 ± 2.35 **
**AP-3** (5 μM)	0.75 ± 0.01 **	51.68 ± 2.18 **	**AP-13** (5 μM)	1.05 ± 0.02 **	87.58 ± 1.60
**AP-3** (10 μM)	0.72 ± 0.01 **	50.92 ± 0.93 **	**AP-13** (10 μM)	1.00 ± 0.02 **	80.55 ± 1.27
**AP-4** (5 μM)	0.65 ± 0.01 **	59.22 ± 1.74 **	**AP-14** (5 μM)	1.36 ± 0.04	54.00 ± 1.27 **
**AP-4** (10 μM)	0.63 ± 0.01 **	56.04 ± 0.71 **	**AP-14** (10 μM)	1.17 ± 0.01 **	49.01 ± 0.25 **
**AP-5** (5 μM)	0.68 ± 0.02 **	71.22 ± 0.81	**AP-15** (5 μM)	1.40 ± 0.03	69.27 ± 1.78 *
**AP-5** (10 μM)	0.62 ± 0.01 **	66.47 ± 0.40 *	**AP-15** (10 μM)	1.32 ± 0.04	67.29 ± 0.87 *
**AP-6** (5 μM)	0.48 ±.0.01 **	41.89 ± 1.21 **	**AP-16** (5 μM)	1.03 ± 0.01 **	64.71 ± 1.98 *
**AP-6** (10 μM)	0.15 ± 0.01 **	19.75 ± 2.14 **	**AP-16** (10 μM)	0.95 ± 0.01 **	57.23 ± 2.05 **
**AP-7** (5 μM)	1.17 ± 0.01 **	75.84 ± 0.20	**AP-17** (5 μM)	1.19 ± 0.01 **	67.02 ± 0.87 *
**AP-7** (10 μM)	1.14 ± 0.01 **	71.28 ± 0.12	**AP-17** (10 μM)	1.01 ± 0.01 **	61.50 ± 2.99 **
**AP-8** (5 μM)	0.43 ± 0.03 **	43.28 ± 1.12 **	**AP-18** (5 μM)	1.02 ± 0.04 **	73.15 ± 0.76
**AP-8** (10 μM)	0.23 ± 0.01 **	30.03 ± 1.14 **	**AP-18** (10 μM)	0.98 ± 0.01 **	69.47 ± 0.47 *
**AP-9** (5 μM)	1.26 ± 0.01 *	77.64 ± 3.35	**AP-19** (5 μM)	1.09 ± 0.04 **	76.53 ± 0.91
**AP-9** (10 μM)	1.17 ± 0.01 **	75.20 ± 1.10	**AP-19** (10 μM)	1.07 ± 0.01 **	71.70 ± 1.09

Data are represented as the mean ± SE of three independent experiments. * *p* < 0.05, ** *p* < 0.01 versus LPS treatment with Student’s *t*-test.

**Table 4:** The actual pI, predicted pI and their residuals of the training and test set molecules.

	IL-6					TNF-α				
Name	Actual	CoMFA		CoMSIA		Actual	CoMFA		CoMSIA	
		Pred	Res	Pred	Res		Pred	Res	Pred	Res
**AP-1** *^#^	6.081	6.150	0.070	6.206	0.126	4.276	4.328	0.052	4.335	0.060
**AP-2** ^#^	6.161	6.150	−0.011	6.206	0.045	4.244	4.328	0.084	4.335	0.092
**AP-3**	6.143	6.042	−0.101	6.116	−0.027	4.145	4.149	0.005	4.289	−0.004
**AP-4**	6.201	6.224	0.024	6.182	−0.018	4.293	4.311	0.018	4.254	0.002
**AP-5** ^#^	6.208	6.368	0.161	6.292	0.084	4.252	4.238	−0.014	4.427	0.250
**AP-6**	6.824	6.661	−0.163	6.674	−0.150	4.178	4.524	0.346	4.706	0.001
**AP-7** *^#^	5.943	6.637	0.694	6.692	0.750	4.704	4.676	−0.028	4.674	0.527
**AP-8**	6.639	6.696	0.058	6.735	0.097	4.147	4.664	0.517	4.522	−0.000
**AP-9**	5.932	5.881	−0.051	5.888	−0.044	4.522	4.551	0.028	4.129	0.005
**AP-10**	6.027	5.990	−0.036	6.019	−0.008	4.124	4.122	−0.002	4.220	0.001
**AP-11**	5.955	5.934	−0.021	5.956	0.001	4.220	4.232	0.013	4.173	−0.002
**AP-12** *	6.092	5.898	−0.194	5.890	−0.202	4.175	4.161	−0.014	4.205	−0.001
**AP-13**	6.000	6.002	0.002	5.985	−0.015	4.206	4.206	0.001	4.112	0.001
**AP-14** ^#^	5.932	5.936	0.004	5.947	0.016	4.110	4.095	−0.015	4.169	−0.141
**AP-15**	5.8780	5.944	0.065	5.943	0.064	4.310	4.170	−0.139	4.169	−0.004
**AP-16** ^#^	6.022	5.989	−0.033	5.974	−0.049	4.172	4.183	0.011	4.152	−0.091
**AP-17** *	6.000	6.057	0.062	6.036	0.040	4.242	4.125	−0.117	4.211	0.000
**AP-18**	6.009	6.036	0.028	5.982	−0.027	4.211	4.206	−0.005	4.159	0.001
**AP-19**	5.971	6.045	0.075	6.001	0.031	4.159	4.161	0.003	4.143	−0.002

* Group 1: Test set molecules (IL-6); ^#^ Group 2: Test set molecules (TNF-α).
